# Clinical and Prognostic Implications of Cervical and Ocular Vestibular Evoked Myogenic Potentials (cVEMP and oVEMP) in Benign Paroxysmal Positional Vertigo (BPPV): A Prospective Study

**DOI:** 10.3390/audiolres13050061

**Published:** 2023-09-12

**Authors:** Maria Silvia Rosa, Massimo Campagnoli, Davide Masnaghetti, Fausto Taranto, Giulia Pisani, Massimiliano Garzaro, Paolo Aluffi Valletti

**Affiliations:** Department of Otorhinolaryngology—Head and Neck Surgery, University of Eastern Piedmont, 28100 Novara, Italy; m.silviarosa@gmail.com (M.S.R.); davide.masna@gmail.com (D.M.); fausto.taranto@maggioreosp.novara.it (F.T.); giulia.pisani@maggioreosp.novara.it (G.P.); massimiliano.garzaro@uniupo.it (M.G.); paolo.aluffi@med.uniupo.it (P.A.V.)

**Keywords:** cervical/ocular vestibular evoked myogenic potentials, cVEMPs, oVEMPs, benign paroxysmal positional vertigo, BPPV, utricular, saccular, ENT, vertigo, dizziness

## Abstract

Objective: Several studies have investigated the efficacy of VEMP (vestibular evoked myogenic potential) in patients with vestibular disorders and BPPV (benign paroxysmal positional vertigo). However, previous data were inconclusive. The aim of this study was to investigate the difference in latency, amplitude P1-N1, asymmetry ratio (AR), and cervical/ocular-VEMP values between BPPV patients and healthy controls. Methods: 125 healthy subjects and 42 BPPV patients were prospectively enrolled in the study. In both groups, c/oVEMP tests with 500 Hz tone-burst stimuli were performed. Latencies P1, N1 peaks, and corrected amplitudes (CA) were measured, and AR was calculated. Results: in the BPPV group, 14.29% of patients lacked oVEMPs that recovered after therapy. N1 latencies were significantly elongated, and 50% of patients had pathological AR; this value normalized at follow-up sessions. In addition, there was a reduction in CA in the pathologic ear compared to healthy ears (*p* = 0.04) and compared to healthy controls (*p* = 0.01). For cVEMP, a significant reduction in latency-P1 was observed in BPPV patients compared to controls; no significant differences were observed for P1, N1, and CA values between the two ears. The cVEMPs were absent in 14.29% of BPPV patients (AR > 35) that recovered after therapy. Conclusion: We identified several abnormal c/oVemp values in BPPV patients compared with healthy controls, with most changes in values occurring in oVEMPs, suggesting that utricular dysfunction may be more common than saccular. In addition, patients with oVEMP alteration showed later clinical recovery, suggesting a possible prognostic role of the test.

## 1. Introduction

Benign paroxysmal positional vertigo (BPPV) is the most common cause of peripheral vertigo [1] and is characterized by brief attacks of vertigo triggered by head movements. A widely accepted theory regarding the pathophysiology of BPPV is the detachment of the otoconia and otoconial debris from the neuroepithelial membrane of the utricular or saccular macula [2,3]. The entry of otoconia into the semicircular canals leads to the onset of symptoms, characterized by intense brief objective vertigo and paroxysmal nystagmus. Because of its anatomic location, the posterior semicircular canal is the site most commonly affected by the pathology. The affected canal can be identified by the direction of nystagmus caused by the movements of the otoliths within the canal [4]. The vestibular evoked myogenic potential (VEMP) is a short-latency myogenic response evoked by brief pulses of air-conducted (AC) sound, bone-conducted (BC) vibration or electrical stimulation and is recorded with surface electrodes placed over muscles. Electrode positioning differs for c and oVEMPs; for cervical VEMPs, an active electrode is located in the cranial third of the sternocleidomastoid muscle, a reference electrode is located at half of the clavicle, and a ground electrode is located in the middle of the forehead. For ocular VEMPs, there are two reference electrodes (one under each eye), an active electrode on the chin, and a ground electrode in the middle of the forehead [5]. Otolith function can be assessed with both ocular (oVEMPs) and cervical (cVEMPs) vestibular evoked myogenic potentials [6]. Cervical VEMPs were first described by Colebatch and Halmagyi in 1992 and 1994, respectively [7,8], whereas oVEMPs were first described by Rosengren and Todd about 10 years later [6]. cVEMPs are known to reflect the function of the ipsilateral sacculo-collic inhibitory pathways [8,9,10], which include a reflex arc of saccule, inferior vestibular nerve, and sternocleidomastoid muscle (SCM). The preferred intensity of the click or tone-burst was above 90 or 100 db (normal hearing level, nHL). The preferred frequency was above 500 Hz [8,9,10,11]. Electromyographic activity was bandpass-filtered, and the resulting response consisted of a biphasic wave with an initial positive peak at 12–13 ms latency [P1] and a subsequent negative peak at 22–23 ms latency (N1). P1 and N1 represent a transient inhibitory and excitatory muscular response, respectively, as a result of the stimulating tone burst [12]. In contrast, the oVEMP recorded from the extraocular muscles likely reflects a manifestation of the crossed utriculo-ocular reflex pathways and features a reflex arc of the utricle, superior vestibular nerve, and extraocular muscles [13,14]. The oVEMP originates in the obliquus inferior muscle and is produced by a brief excitation of the muscle The response consists of a biphasic surface potential with peaks at approximately 10 and 15 ms, beginning with a negativity [15].

To our knowledge, several articles have investigated the efficacy of VEMPs in patients with BPPV. Most studies in the literature considered both cVEMP and oVEMP values to compare patients affected by BPPV to healthy controls. In all cases, a significant difference in c and oVEMP values was found between BPPV groups and healthy control subjects [16,17,18]. Very few data are available on the differences in recording VEMPS in patients affected by recurrent BPPV. In particular, Hui Xu et al. described that in their population, oVEMP abnormalities were significantly higher in the recurrent BPPV group than in the non-recurrent BPPV group [19]. The aims of this study included: (i) testing the difference in latency P1, N1, and the asymmetry ratio (AR) in c/o-VEMPs between patients with BPPV and controls, (ii) testing the difference between oVEMPs and cVEMPs in BPPV patients, and (iii) evaluating the prognostic potential of the test.

## 2. Materials and Methods

### 2.1. Subjects

This prospective observational study was conducted between February and September 2021. First, we performed c and oVEMPs on a large sample of healthy controls (n = 125). The sample was representative in terms of age and gender distribution to establish our normal values in terms of P1 and N1 latency and amplitude.

The BPPV group was selected considering the following inclusion criteria:

- Patients with first diagnosis of monolateral BPPV
- No history of otologic pathology
- Age of the patients between 18 and 65 years
- Informed consent agreement

No selection was made based on the involved canal; all 5 types of BPPV described by Ichijo were included in the study [20].

Forty-two patients with first diagnosis of BPPV were subsequently enrolled in the study. The diagnosis of BPPV and the affected side were based on clinical history and typical nystagmus observed during the Dix–Hallpike and Pagnini–McClure maneuvers. Thirty patients had posterior canal BPPV, and 12 patients had lateral canal BPPV.

At the first visit, a history was taken to asses vestibular symptoms (type and duration of vertigo), auditory symptoms (presence of subjective hearing loss, tinnitus, ear fullness), previous ENT department procedures, and presence of risk factors for the development of the pathology in question (head injury in the months preceding the vertigo attack; drug or ototoxic substance intake; neurosurgical or otosurgical procedures; and infectious diseases such as viral otitis, osteoporosis on vitamin D3 therapy, previous biliary, or renal lithiasis).

All participants underwent neurotologic examination, pure tone audiometry, and bilateral cVEMP and oVEMP recording before therapy with repositioning maneuvers (pre-therapy), 48 h after recovery (post-therapy), at 1 month, and at 3 months after diagnosis.

Patients were treated with the Epley’s maneuver if the posterior semicircular canal was affected, or with the Gufoni repositioning maneuver if the lateral semicircular canal was involved. The presence of nystagmus during the diagnostic maneuver was assessed clinically. All patients were readmitted after 2 days to determine whether the repositioning maneuver was effective in resolving BPPV. All patients affected by BPPV in the lateral canal were advised to sleep on the side on which they had fewer symptoms. This routine was carried out until the patients recovered. The patient was considered recovered if no nystagmus occurred during diagnostic maneuvers. Exclusion criteria included neurologic and/or otologic disorders, transmission hearing loss documented by pure tone audiometry, and history of recurrent BPPV. In addition, patients older than 65 years old were excluded due to a higher incidence of VEMP abnormalities in this population.

Patients had a mean age of 51.2 ± 11.3 (*p* = 0.43). Six of the 42 patients were male (14.29%), and the rest were female (85.71%) (*p* = 0.11). There were no cases of bilateral BPPV in our cohort. There was no statistically significant age difference between the patients and the control group. Thirty-one patients were diagnosed with posterior canal BPPV. The remaining 11 patients had VPPB of the lateral semicircular canal; 7 of them with the geotropic variant and four with the apogeotropic.

In accordance with the Declaration of Helsinki, oral and written informed consent was obtained from all participants.

### 2.2. VEMPs

A VEMP potential system (Neuro-Audio, Neurosoft made in Russia) was used to record cervical and ocular VEMPs.

For cVEMPs, disposable silver chloride electrodes (Neuro-Audio, Neurosoft made in Russia) with an impedance of ≤5 kΩ were used, while for oVEMPs, clip-electrodes (SpesMedica, model Denis 02025—20 × 25 mm Snap) with an impedance of ≤5 kΩ were used. An EMG feedback system (Neuro-Audio, Neurosoft made in Russia) was used for recording muscle responses. EMG was amplified (100 dB) and band pass filtered (30–2000 Hz for oVEMPs and 1–1000 Hz for cVEMPs). Muscle responses were recorded 5 ms before the stimulus onset to 50 ms after stimulus onset. To improve reliability and reduce interpatient variability, the test was repeated three times.

### 2.3. cVEMP Recording

Recordings were performed in the upright sitting position. The active electrode was positioned over the midpoint of the SCM muscle. The reference electrode was placed on the sternum. A ground electrode was placed on the forehead. Participants were asked to turn their heads contralateral to the stimulated ear and to tilt their head slightly forward to achieve sufficient muscle contraction.

Through headphones, air-conducted sound (ACS) 100 dB nHL tone pulses were presented at 500 Hz, and the EMG signal was recorded.

We analyzed the waveforms of P1 and N1 at the maximum stimulation intensity. The amplitude of the first positive–negative peak (P1–N1) was recorded. The absence of a meaningful waveform at P1 and N1 was defined as “no response”. The latencies of P1 and N1 peaks and the normalized P1 and N1 amplitudes were measured for the 500 Hz frequency. Because background muscle activity can interfere with VEMP amplitudes, amplitudes were normalized by dividing raw amplitudes by background EMG activity (corrected amplitude, CA).

The AR was calculated to compare the right and left ears, using the formula described by Murofushi et al.; a value greater than 35 is considered pathological [14].

Asymmetry Ratio (AR%) = 100(Au − Aa)/(Au + Aa)

Au: p1-n1 (the peak-to-peak amplitude of the unaffected ear)

Aa: p1-n1 (the peak-to-peak amplitude of the affected ear)

### 2.4. oVEMP Recording

The oVEMPs were performed with subjects sitting upright and looking superomedially at a small, fixed target 1 m from their eyes. The visual angle was approximately 30°, which elicited the largest responses compared to other eye positions [19]. The active electrodes were placed approximately 1 cm below the center of the lower eyelid just below the contralateral eye for the tone stimulation. The reference electrode was placed approximately 1 cm below the active electrode on the cheek, and the ground electrode was placed on the forehead. An air-conducted sound (ACS) 100 dB nHL tone at 500 Hz was presented through headphones, and the EMG signal was recorded. We analyzed the waveforms of N1 and P1 at the maximum stimulation intensity.

### 2.5. Statistical Analysis

To check normal distribution and homogeneity of variance in the groups, Shapiro–Wilk and Levene tests were performed.

For categorical results, Fisher’s exact test and the chi-squared test were used. The *t*-test and analysis of variation (one-tailed ANOVA) were performed to analyze the differences between groups and between the parameters in c and oVEMPs in BPPV patients and controls in terms of continuous outcomes. A *p*-value ≤ 0.05 indicates statistical significance. In order to clarify data that are immediately comprehensible, box plots were created.

## 3. Results

### 3.1. Healthy Controls

Data from a total of 125 healthy control subjects were analyzed to define the normal range of the main parameters of oVEMP and cVEMP, such as the latencies of P1 and N1, the corrected amplitude (CA), and AR. The control group consisted of patients who visited our ENT practices and had neither vertigo, nor dizziness, nor otologic pathology.

The mean age of the healthy control subjects was 47 ± 12.4 years. There were 33 males (26.4%) and 92 females (73.6%).

No statistically significant differences (*p*-value = 0.11) were found between gender in any of the parameters studied. A decrease in the amplitude of the potentials was observed with increasing age; in particular, a statistically significant reduction was observed after the age of 50 (*p*-value = 0.03). The results of this analysis are presented in Table 1 and Table 2.

### 3.2. oVEMPs

Among the 42 patients, only 6 (14.29%) had no oVEMPs on the pathological side, while the remaining 85.71% presented VEMPs. However, in the cases in which oVEMPS were absent, oVEMPs became identifiable during follow-up; in 50%, immediately after treatment, while in the other 50%, after 3 months.

**Figure 1:**
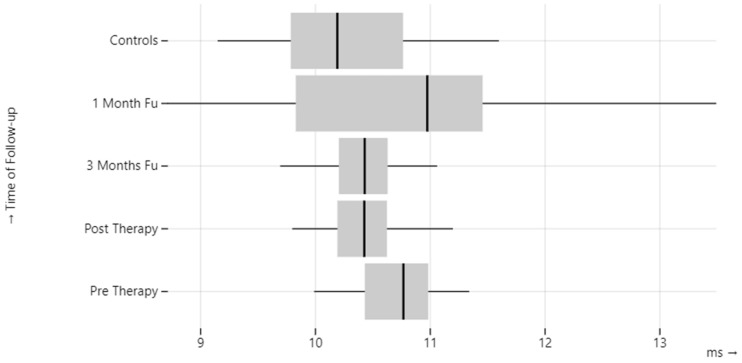
oVEMP N1 latency in BPPV patients.

N1 latencies were significantly prolonged in BPPV-affected ears compared to healthy controls group values post-treatment (*p*-value < 0.01), at the 1-month (*p*-value = 0.01) and at the 3-month follow-up (*p*-value = 0.04) examination. In the unaffected ears, N1 latencies were increased compared to controls (*p*-value = 0.01) (Table 3, Figure 1).

On examination before therapy, 21/42 patients (50%) had pathologic interaural asymmetry (AR > 35). Asymmetry returned to the normal range in 51.14% of patients after therapy, and in 14.29%, at the three-month follow-up, while it remained pathologic in 28.57% at all follow-up examinations.

### 3.3. cVEMPs

In 14.29% of patients, cVEMP was absent on the pathological side before treatment, (AR > 35),which recovered completely when examined after treatment.

There were no significant differences in P1, N1, and CA values between the pathological and the healthy ears.

**Figure 2:**
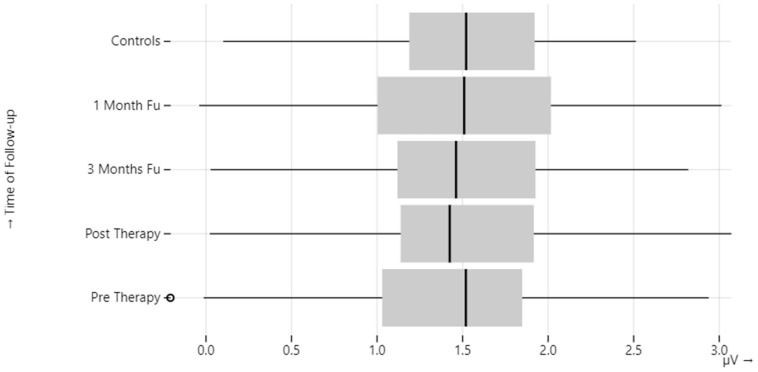
cVEMP corrected amplitude (CA) in BPPV patients.

**Figure 3:**
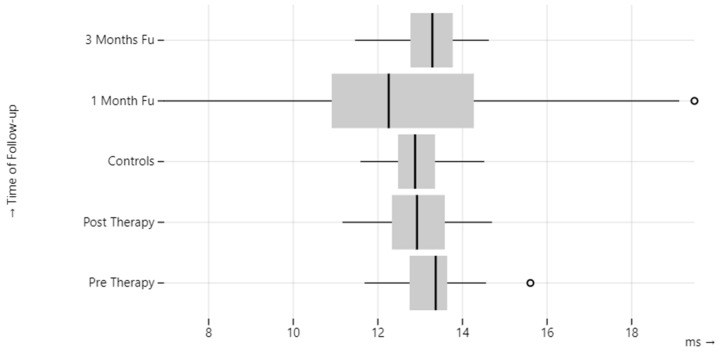
cVEMP P1 latency in BPPV patients.

Compared to the healthy control group, a statistically significant difference (*p*-value = 0.01) was observed in terms of one-month CA on the pathological side, latency of P1 before therapy, and CA after therapy on the healthy side (Table 4, Figure 2 and Figure 3).

In the present case, only three patients showed a recurrence of BPPV on the opposite side from the first episode. For this reason, it was not possible to perform a comparison between relapsing and non-relapsing patients.

## 4. Discussion

Although BPPV is the most common cause of peripheral vertigo, the exact pathophysiology remains unclear. Several studies have suggested that the cause may be a dislodgement or detachment of the otoconia from the otolith membrane [17,20,21], which may be associated with osteopenia and osteoporosis [14]. BPPV patients usually respond well to treatment, but there is a significant relapse rate after the initial resolution [19,22]. Recurrence rates have been reported to range from 10% to 18% [23,24,25].

cVEMPs and oVEMPs are now commonly used to test otolith function in patients with vertigo and balance disorders [25]. They are used to demonstrate loss of otolith function in the presence of damage to the inner ear, vestibular nerve, or central vestibular pathways.

The literature indicates that normal values vary significantly among different healthcare centers. Therefore, it is recommended that each center establishes its own normal values. First, we examined a sample of healthy patients to delineate the normal values in our center. We determined that the normal values of oVEMPs are consistent with those in the literature, whereas the values of cVEMPs showed a slightly lower latency [5].

For both oVEMPs and cVEMPs, there was a decrease in amplitude with increasing age, especially above 50 years, while there was no correlation with gender; these data are confirmed by the literature [25].

In the present study, both cVEMPs and oVEMPs were used to assess saccular and utricular function in patients with BPPV before the repositioning maneuvers and 48 h, 1 month, and 3 months later. In our study, 14.29% of patients had no cVEMP on the pathological side before treatment, which recovered completely during the follow-up period. No significant differences in P1, N1, and CA values were found between the pathologic and healthy ears.

In a study conducted by Hong et al. on patients with BPPV, 24.5% of patients had abnormalities on the pathological side in cVEMP tracing, such as increased P1 or N1 latency or pathological interaural AR [17].

In another study conducted by Karataş et al., no differences in latency or AR were found in patients with BPPV compared with healthy controls. However, a reduction in the amplitude of cVEMP was noted in both pathological and healthy ears [26].

Xu et al. examined the characteristics of cVEMP and oVEMP in patients with BPPV and found that a greater number of changes occurred on the pathological side compared with healthy controls. Indeed, cVEMPs were altered in 30% of cases, whereas oVEMPs were altered in 56.7%.

Moreover, with regard to cVEMP, there were no statistically significant differences between patients with recurrent and non-recurrent BPPV. In contrast, 90% of patients with recurrent BPPV had altered oVEMP compared with 40% of patients with non-recurrent BPPV. The authors suggested that damage to the otolithic organs and, in particular, of the utricle, underlies the pathology and that VEMPs may be useful in assessing the risk of disease recurrence [18].

In agreement with the works cited above, we also found significant differences in oVEMP and cVEMP between the pathological ear and the healthy ear. The statistically significant difference in oVEMPs relates particularly to N1 latency, which was long in the test before therapy. For the cVEMPs, a significant reduction was observed during the follow-up one month after the maneuvers at CA values. These results suggest that the disease affects the entire vestibule, involving the utricle in the early stages and the saccule later. Compared with healthy controls, patients showed increased P1 latency before therapy and decreased latency after therapy in the healthy ear.

In addition, 50% of the patients showed pathological AR on the oVEMP and 14.29% of patients on the cVEMP at initial evaluation. These data also seem to confirm that in BPPV, the utricle is more affected than the saccule. All patients with pathological AR on cVEMP showed normalization of the parameter within three months, while 28.57% of patients with pathological AR on the oVEMP showed no improvement in asymmetry.

These patients still complained of a feeling of instability after three months. Therefore, we hypothesized that patients with greater involvement of the utricle and superior vestibular nerve as measured by the oVEMP, would have slower recovery of vestibular function, as evidenced by longer-lasting symptoms. In these patients, vestibular rehabilitation could play an important role, as it has been shown to improve patients’ balance. As evidence of its efficacy, Bressi et al. have described how the canalith repositioning procedure and vestibular rehabilitation seem to have a synergic effect in patients with BPPV, especially in elderly patients. Vestibular rehabilitation does not reduce the recurrence rate, but it seems to reduce discomfort. Thus, vestibular rehabilitation may substitute the canalith repositioning procedure when spinal comorbidities contraindicate the canalith repositioning procedure, and it may reduce the use of anti-vertigo drugs post canalith repositioning procedure [27,28].

As in Karatas et al., cVEMPs values in our study did not show significant differences between the two ears. This could be due to the lower degree of saccule involvement in the pathology or to the alterations also present at the level of the healthy ear [26].

In the case study investigated, only three patients showed a relapse of BPPV on the same side compared to the first episode. In the pre-therapy examination performed in the first episode, the cVEMP showed an increase in the CA compared to the normal range in both ears, but more evident at the level of the healthy ear. The latency of P1 and N1 was also more increased in the healthy ear. In subsequent controls before relapse, the amplitudes of the cVEMPs remained elevated and also showed an increase of CA on the pathological side. Amplitudes remained bilaterally high in both examinations even after relapse. None of the other patients showed a persistent bilateral increase in CA. These results suggest the presence of persistent alterations at both the utricle and the saccule levels that favor detachment of otoliths. Therefore, the presence of abnormalities of the VEMPs detected in several patients could be predictive of a greater risk of disease recurrence.

A larger sample of BPPV patients should be examined to provide sufficient evidence for a statistical test of whether abnormalities of the VEMPs predict a greater risk of disease recurrence.

## 5. Conclusions

The role of VEMPs in the diagnosis, prognosis, and follow-up of various vestibular pathologies is not yet clear, and the literature remains inconclusive. In our study, 14.29% of 42 BPPV patients had missing oVEMPs and cVEMPs on the pathological side. However, in all cases in which these were absent, they became identifiable during follow-up. Both oVEMPs and cVEMPs were found to have altered values of the parameters studied. On pre-therapy examination, 50% had pathologic interaural asymmetry (AR > 35), which returned to normal in 51.14% of the patients immediately after therapy but was still pathologic in 28.57% after 3-months follow-up. This may suggest that patients with greater utricle and superior vestibular nerve involvement, as measured by oVEMP, have a slower recovery of vestibular function, as evidenced by longer-lasting symptomatology. The presence of persistent alterations at the level of both the utricle and the saccule, identified in recurrent BPPV, could indicate a predisposition to otolith detachment. Therefore, the presence of abnormalities of the VEMPs detected in multiple patients could predict a greater risk of recurrence. Further studies should be conducted to investigate a possible correlation between altered oVEMP/cVEMP values and relapsing patients.

## Figures and Tables

**Table 1:** oVemp normal “healthy controls” values.

oVEMP	
Latency N1 (ms)	10.23 ± 0.67
Latency P1 (ms)	15.07 ± 1.12

**Table 2:** cVemp normal “healthy controls” values.

cVEMP	
Latency P1 (ms)	12.96 ± 0.67
Latency N1 (ms)	20.80 ± 1.21
Corrected amplitude (µV)	1.56 ± 0.52

**Table 3:** oVEMP N1 latency in BPPV patients.

		BPPV-Affected Ear	BPPV-Unaffected Ear	Control Group
N1 (ms)	Pre-therapy	10.74 ± 0.37	10.50 ± 0.61	10.23 ± 0.67
	Post-therapy	10.42 ± 0.31	10.37 ± 0.53	
	1 month	10.84 ± 1.18	10.61 ± 0.38	
	3 months	10.38 ± 0.32	10.52 ± 0.69	

**Table 4:** cVEMP corrected amplitude (CA) and P1 latency in BPPV patients.

		BPPV-Affected Ear	BPPV-Unaffected Ear	Control Group
Ca (µV)	Pre-therapy	1.49 ± 0.68	1.62 ± 0.73	1.56 ± 0.52
	Post-therapy	1.47 ± 0.60	1.64 ± 0.75	
	1 month	1.56 ± 0.72	1.81 ± 0.82	
	3 months	1.51 ± 0.63	1.77 ± 0.90	
P1 (ms)	Pre-therapy	13.25 ± 0.74	13.66 ± 1.15	12.96 ± 0.67
	Post-therapy	13.01 ± 0.85	13.14 ± 1.04	
	1 month	12.71 ± 2.81	13.29 ± 1.34	
	3 months	13.31 ± 0.75	13.03 ± 0.70	

## Data Availability

Data will be available on request.
